# Recognition of Vertebral Artery Dissection in a High-Risk Postpartum Patient by a Chiropractic Physician

**DOI:** 10.7759/cureus.90389

**Published:** 2025-08-18

**Authors:** James Demetrious, Robert J Trager, Stephen Veigh, Peter Tuchin, David Graber

**Affiliations:** 1 Chiropractic, PostGradDC, Wilmington, USA; 2 Family Medicine and Community Health, Case Western Reserve University School of Medicine, Cleveland, USA; 3 Chiropractic, Connor Whole Health, University Hospitals Cleveland Medical Center, Cleveland, USA; 4 Neuroradiology, Private Practice, Sarasota, USA; 5 Chiropractic, Private Practice, Sydney, AUS; 6 Chiropractic, Private Practice, Parsippany, USA

**Keywords:** cervical artery dissection, connective tissue disorders, magnetic resonance angiography, neurovascular imaging, postpartum, preeclampsia, stroke, vertebral artery dissection

## Abstract

We report a case of vertebral artery dissection (VAD) in a patient with postpartum preeclampsia and underscore the contribution of chiropractic assessment in recognizing vascular pathology, facilitating neurovascular imaging, and expediting emergency intervention. A 36-year-old woman with a history of mixed connective tissue disease presented to the emergency department five days postpartum with facial and peripheral edema, recent onset of hypertension, acute severe neck pain, headache, lightheadedness, confusion, and gait instability. Magnetic resonance angiography (MRA) and magnetic resonance venography of the head were performed. Small, unruptured bilateral internal carotid artery aneurysms were visualized. The patient was admitted and treated for postpartum preeclampsia. Upon discharge, she followed up with a neuroendovascular specialist. Although the patient improved with preeclampsia management, concerning neurologic symptoms persisted. The patient later consulted a chiropractor, who suspected VAD. Emergent neck MRA was recommended and performed, confirming VAD 12 days after the initial emergency department visit. The patient was initiated on antithrombotic therapy, with subsequent resolution of symptoms and no evidence of ischemic stroke. This case underscores the importance of maintaining a high index of suspicion for VAD in postpartum patients presenting with new-onset neurological symptoms, particularly in the context of hypertensive disorders of pregnancy and underlying connective tissue disease. It further illustrates the potential role of chiropractic evaluation in the early clinical recognition of vascular pathology, which may facilitate timely diagnosis and intervention, and reduce the risk of stroke.

## Introduction

Vertebral artery dissection (VAD) is a type of cervical artery dissection (CeAD) involving a tear in the wall of the vertebral artery, resulting in hematoma formation. It may cause ischemic stroke, particularly in young adults, and has an incidence rate of approximately two per 100,000 person-years [1]. The clinical presentation of VAD may include occipital headache, neck pain, and other potential features such as visual disturbances, dizziness, and gait abnormalities [2] that can be indicative of brain ischemia. Risk factors for VAD are increasingly understood, such as hypertension, migraine, and connective tissue disorders [3-5].

There is often a delay in the diagnosis of CeAD, with one study reporting a mean time to diagnosis for VAD of 9.8 days (standard deviation of 13.5 days) [6]. CeAD may also be misdiagnosed in the emergency department, with rates of misdiagnosis estimated from 1.2% to 5.4% [7]. Potential reasons for misdiagnoses or delays of VAD include the potentially nonspecific and variable clinical presentation, a low index of suspicion in young patients, cognitive error (e.g., anchoring, premature closure), and diagnostic overshadowing by more common conditions (e.g., migraine, musculoskeletal neck pain, or tension headache) [2,6,7].

Pregnancy is an increasingly recognized risk factor for CeAD and VAD. One study reported that pregnant women had approximately two times the risk of VAD compared to non-pregnant controls, with an elevated incidence of VAD during a one-year window including pregnancy and postpartum of 5.9 (95% confidence intervals [CI]: 3.7-8.1) per 100,000 person-years [8]. Similarly, a case-control study identified a two-fold increase in CeAD risk associated with pregnancy [9]. In that study, the risk of CeAD was greatest during the postpartum period, with an incidence rate ratio of 5.5 (95% CI: 2.6-11.7) [9]. Despite this increased risk, CeAD and VAD may be particularly underrecognized in postpartum women, given the nonspecific symptoms such as headache and neck pain [10].

Patients often present to chiropractic physicians for evaluation of head and neck pain. Accordingly, these clinicians may be positioned to identify red flags suggestive of vascular pathology [11]. In this case report, we describe a postpartum patient whose diagnosis of VAD was delayed until chiropractic consultation prompted appropriate vascular imaging. This case highlights the essential role that chiropractic providers can play in the early recognition of potentially disabling neurovascular conditions.

## Case presentation

Initial emergency department (ED) visit (day zero)

A 36-year-old woman, five days postpartum following an uncomplicated spontaneous vaginal delivery, presented to the emergency department (ED) with a two-day history of progressively worsening symptoms. She noted the onset of facial and bilateral upper and lower extremity edema, accompanied by elevated home blood pressure measurements persistently exceeding 140/90 mmHg. She also described the atraumatic development of a band-like temporal headache, photophobia, blurred vision, dizziness, and confusion that are possible manifestations of VAD.

On the day she sought ED care, her clinical condition rapidly deteriorated. She developed a severe occipital headache and new, excruciating, left-sided posterior neck pain with radiation into the left shoulder. The pain significantly restricted active cervical rotation bilaterally. Visual disturbances progressed to include diplopia and intermittent scotomata described as "seeing spots," along with worsening photophobia. Additionally, the patient experienced dyspnea, lightheadedness, and progressive bilateral lower extremity edema that impaired ambulation. She expressed marked anxiety related to the severity and rapid progression of her symptoms.

According to her mother, who accompanied her to the ED, the patient was notably disoriented. Due to the escalation of her symptoms, emergent evaluation was pursued. Initial vital signs and laboratory values were acquired at the ED (Table 1).

**Table 1:** Clinical data table. mmHg: Millimeters of mercury, C: Celsius, g/dL: Grams per deciliter, IU/L: International Units per liter, L: Liter.

Test	Result	Reference Range
Vital Signs		
Blood Pressure	150/103 mm HG	Normal: <120/<80 mm HG
Temperature	36.9° C	36.1-37.2°C
Oxygen Saturation	97% on ambient air	≥ 95% on ambient air
Neurologic Status	Alert and oriented; no focal deficits	Alert and oriented x 3; no deficits
Lower Extremity Edema	2+ bilateral	No edema
Lab Values		
Hemoglobin	10.7 g/dL	Female: 12.1-15.1 g/dL
Platelet Count	188 x 10^9^/L	150-450 x 10^9^/L
Aspartate Aminotransferase	50 IU/L	10-40 IU/L
Alanine Aminotransferase	52 IU/L	7-56 IU/L
Lactate Dehydrogenase	313 IU/L	140-280 IU/L
Albumin	2.8 g/dL	3.5-5.0 g/dL

Magnetic resonance venography of the brain demonstrated a T1 hypointense filling defect in the superior sagittal sinus, interpreted as a benign arachnoid granulation, without evidence of venous thrombosis. Chest radiography was unremarkable. In the context of recent childbirth, elevated blood pressure, peripheral edema, and the above laboratory abnormalities, the patient was diagnosed with postpartum preeclampsia and admitted to the hospital for further management.

Inpatient care (days zero to three)

The patient was treated in an inpatient setting for three days. Initial treatment included a 24-hour infusion of intravenous magnesium sulfate under close monitoring. A neurology consultation was obtained. On inpatient day two, magnetic resonance angiography (MRA) of the head was performed, revealing small, bilateral unruptured paraclinoid internal carotid artery (ICA) aneurysms measuring 0.4 × 0.3 cm on the right and 0.4 × 0.4 cm on the left.

Notably, despite clear indications for neck MRA, the study was not performed, and VAD remained unrecognized and undiagnosed throughout the hospital admission. The patient was discharged with a five-day course of furosemide and a prescription for sustained-release nifedipine 60 mg daily, which she began the day after discharge. She was referred for outpatient follow-up with a neurovascular surgeon.

Neuroendovascular consultation (day seven)

Seven days after the initial ED visit, the patient participated in a video consultation with a neurointerventional surgeon, who reviewed the ICA aneurysms and advised close monitoring for signs of aneurysmal rupture. She was considered an appropriate candidate for staged endovascular treatment involving the placement of flow-diverting stents several weeks apart. However, VAD was not suspected during this evaluation, and no additional imaging was ordered.

Chiropractic consultation (day 11)

Eleven days following her initial presentation to the emergency department, the patient participated in a telemedicine consultation with her former chiropractic physician based out of state. During the consultation, the patient and her mother provided a timeline of her symptom progression. They described an initial cluster of symptoms consistent with preeclampsia, followed by the sudden onset of severe, atraumatic posterior cervical and head pain that was accompanied by visual disturbances, lightheadedness, transient gait instability, cognitive clouding, and a pervasive sense of unease.

The patient reported that although her acute symptoms had gradually improved during preeclampsia-directed care, several residual complaints remained. The previously severe posterior cervical pain had notably abated; however, she continued to experience a persistent, albeit less intense, occipital headache. Lingering symptoms included cognitive clouding, right facial numbness localized to the maxillary distribution of the trigeminal nerve, and paresthesias affecting the right hand.

Upon questioning, the patient reported a past diagnosis of systemic lupus erythematosus that was later refined as a mixed connective tissue disease, diagnosed by a rheumatologist eight years prior, given the presence of positive anti-ribonucleoprotein antibodies, arthralgia, Raynaud phenomenon, and intermittent dysphagia. The patient elected not to pursue immunosuppressive therapy or follow-up rheumatologic care.

The patient also reported a remote history of migraine without aura during adolescence. She denied a recent history of infection, past use of fluoroquinolones, heritable or acquired coagulopathies, anticoagulant medications, arteriopathy, or tobacco, alcohol, or illicit substance use. She was unaware of whether she had fibromuscular dysplasia, patent foramen ovale, or other possible causes of thromboembolism. She had a family history of hypothyroidism (mother) and asthma (father), without a familial history of cerebrovascular disease.

During a prenatal chiropractic wellness visit, approximately four weeks prior to the headache and neck pain onset, the patient received high-velocity, low-amplitude spinal manipulative therapy to the cervical and lumbar spine. She reported no neck pain, headache, or other symptoms preceding or following the intervention.

Considering the constellation of symptoms suggestive of posterior circulation ischemia, the consulting chiropractor suspected VAD, vessel occlusion, pseudoaneurysm, and/or thromboembolism, and thus recommended emergent medical care. He advised the patient and her family to return to the hospital immediately. The chiropractor specifically recommended that the patient obtain MRA or computed tomography angiography of the neck, with a presumptive diagnosis of VAD.

The patient contacted her primary care physician with these concerns, and the physician ordered an MRA of the neck without and with contrast, which was performed at a local private medical facility later that afternoon, 11 days after the initial ED visit.

An attending neuroradiologist read the MRA study of the neck. The imaging protocol included an unenhanced two-dimensional time-of-flight axial MRA with multiplanar reformations, supplemented by an unenhanced axial T1-weighted fat-saturated sequence. Additionally, contrast-enhanced coronal MRA of the neck with multiplanar reformations was obtained. The neuroradiologist noted that an irregular contour was observed throughout the majority of the V2 segment of the left vertebral artery. On axial T1-weighted fat-saturated images, a crescentic intrinsic hyperintense signal was noted surrounding this segment, consistent with intramural hematoma, diagnostic of arterial dissection. The radiologist’s report reflected a long segment dissection of the left vertebral artery of the V2 segment, without evidence of hemodynamic compromise.

The radiologist entered a critical alert using an actionable findings communication system. However, the patient did not receive any communication regarding the finding of VAD and returned home.

Second ED visit (day 12)

Twelve days after the initial ED visit, and 17 days postpartum, the patient returned to the ED with recurrence of severe left-sided neck pain with extension to her left shoulder, pressure and band-like bi-temporal headache, chest pain, and bilateral tingling in the hands and feet, without any identifiable triggers. Her neurological examination was non-focal. The stroke neurology team was consulted and ordered another MRA of the neck, which reconfirmed VAD affecting the left vertebral artery.

Following clearance under the Brain Attack Team, anti-thrombotic therapy was initiated with 325 mg aspirin daily. Given the lack of overt neurologic deficits, the stroke neurologist opted against additional brain imaging or interventions.

Follow-up (day 31)

**Figure 1:**
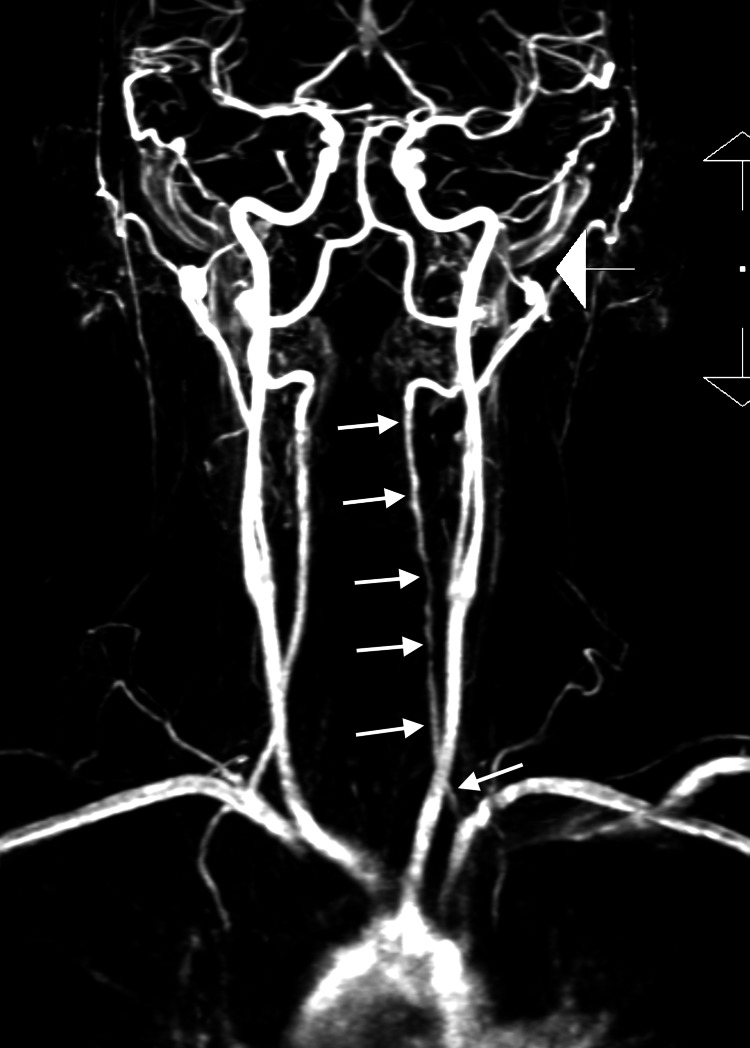
Post-contrast high-resolution coronal magnetic resonance angiography sequence. This shows a long hypointense signal abnormality located in the V2 segment of the left vertebral artery, contributing to varying degrees of significant luminal narrowing consistent with vertebral artery dissection (white arrows).

**Figure 2:**
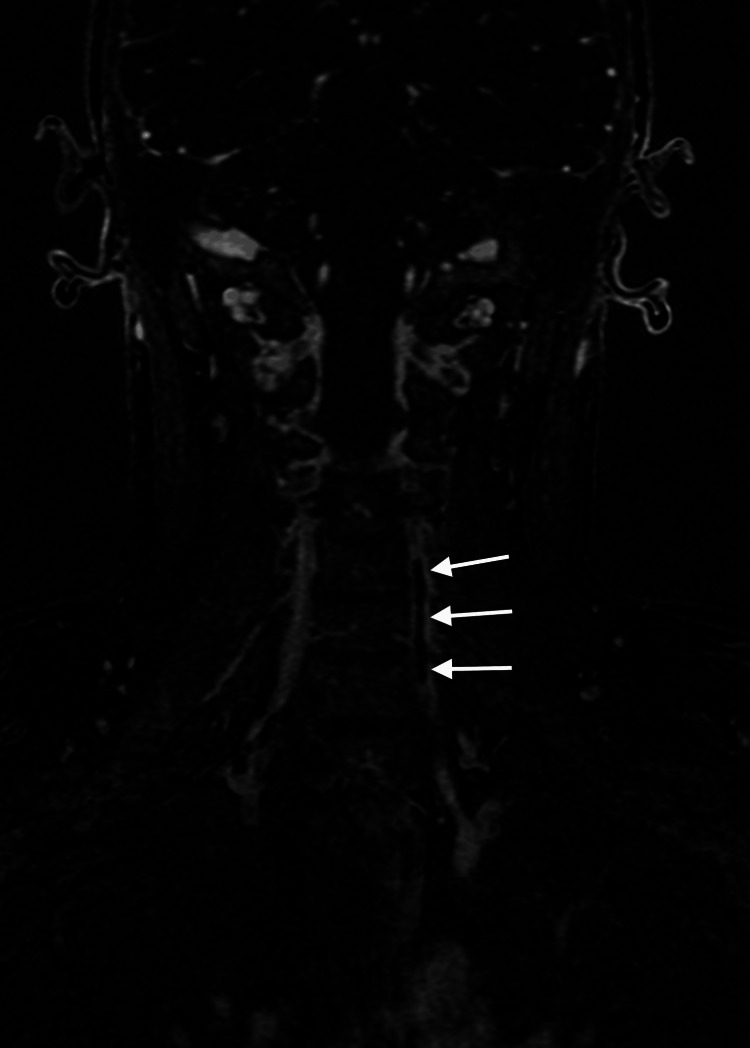
Post-contrast high-resolution coronal magnetic resonance angiographic imaging sequence. This shows an intra-arterial filling defect located in the V2 segment of the left vertebral artery, consistent with VAD (white arrows).

**Figure 3:**
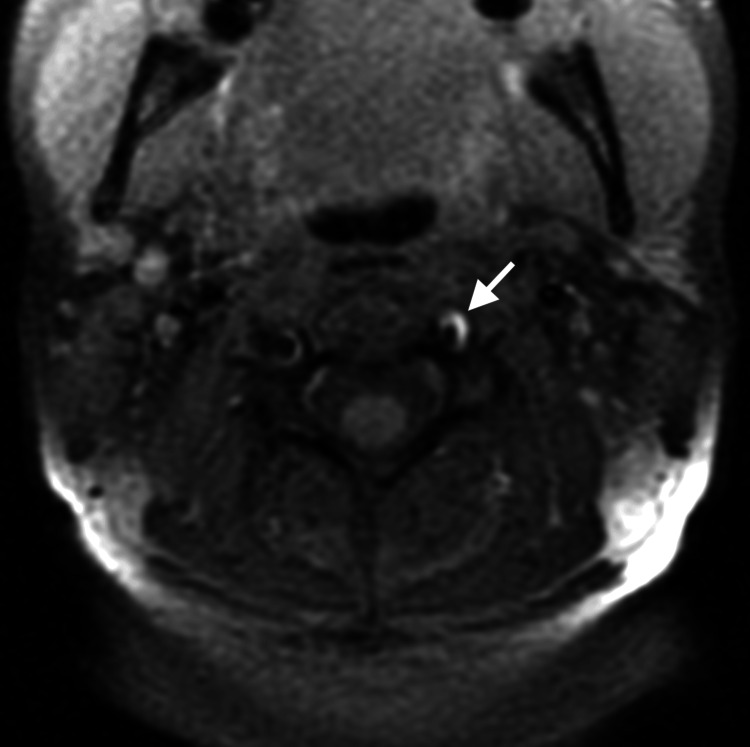
Axial T1-weighted fat-saturated non-contrast magnetization-prepared rapid gradient echo (MPRAGE) image. This shows a hyperintense crescent sign in the V2 segment of the left vertebral artery (white arrow), indicating an acute intramural hematoma within the arterial wall. There is approximately 70–80% stenosis of the arterial lumen present.

**Figure 4:**
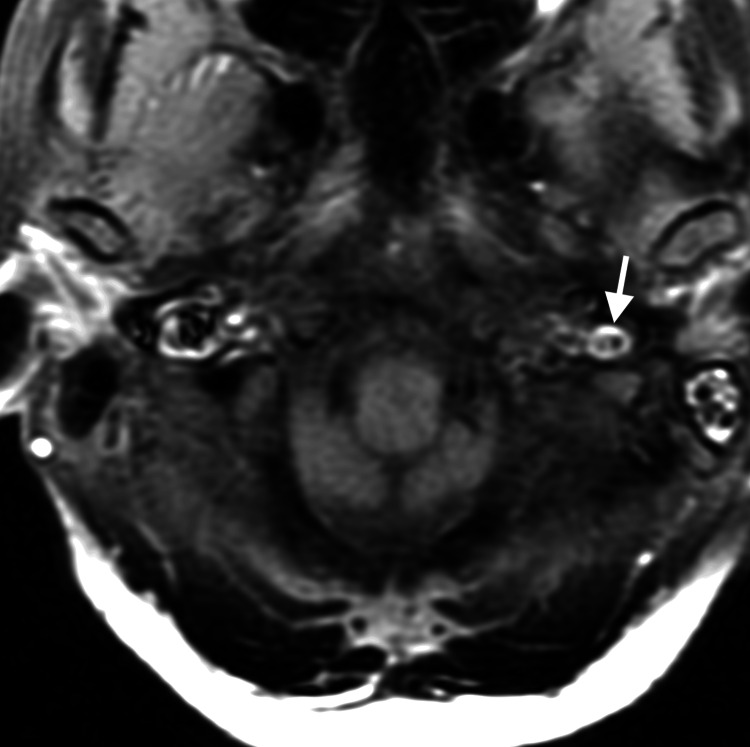
Axial T1-weighted fat-saturated non-contrast magnetization-prepared rapid gradient echo (MPRAGE) image. This shows a hyperintense crescent sign surrounding the V3 segment of the left vertebral artery with central true and false lumens (white arrow), indicating an acute intramural vertebral artery dissection based on differential arterial flow dynamics.

At the request of the attending out-of-state chiropractor, an independent review of the MRA studies was subsequently performed by a fellowship-trained neuroradiologist, who provided additional interpretive findings. The neuroradiologist confirmed the presence of a large, long-segment dissection of the left vertebral artery extending from the origin at the aortic arch through the V3 segment (Figure 1). An associated intramural hematoma was visualized with an intra-arterial filling defect located in the V2 segment of the left vertebral artery (Figure 2). Luminal narrowing of approximately 70-80% was noted, with hyperintense signal characteristics of the intramural hematoma (Figure 3), consistent with an early subacute-late subacute phase, suggestive of a dissection occurring within the preceding 14 days [12]. At the V3 segment, both a central lumen and a false lumen were visualized, indicative of active intramural dissection with differential arterial flow dynamics (Figure 4).

**Figure 5:**
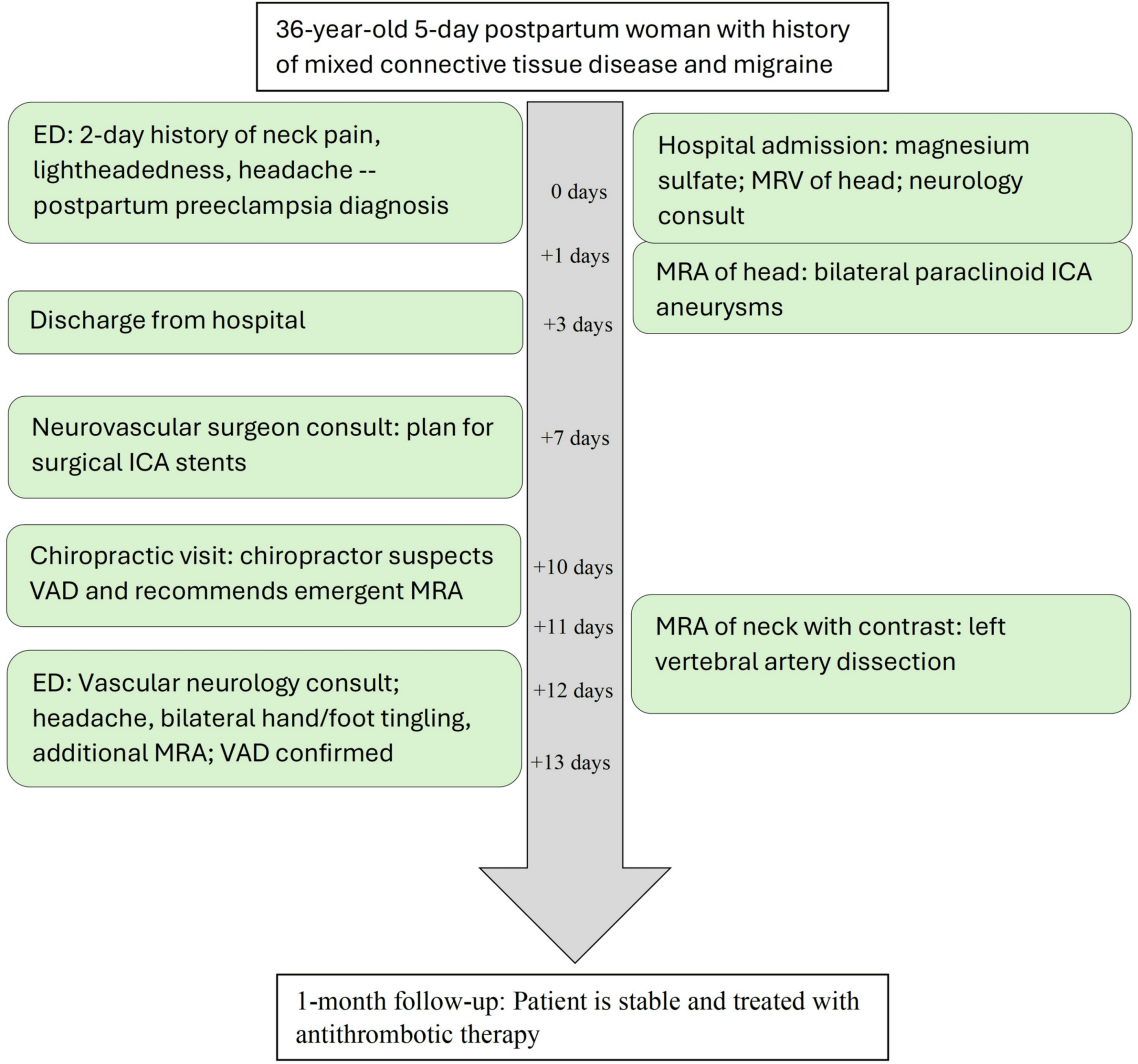
Timeline of events. Dates are shown in relation to the initial emergency visit, rather than calendar dates, for de-identification purposes. VAD: Vertebral artery dissection, ICA: Internal carotid artery, ED: Emergency department, MRV: Magnetic resonance venography, MRA: magnetic resonance angiography.

These detailed imaging findings were thoroughly discussed with the patient. One month later, the patient remained stable and was scheduled for follow-up with both a neurologist and a neurovascular surgeon. The chiropractor recommended further rheumatologic and genetic testing to refine the diagnosis and evaluate for a potential heritable connective tissue disorder and predispositions for recurrent dissections. The timeline of the case is summarized in Figure 5.

## Discussion

This case report presents a 36-year-old woman who was initially diagnosed and treated for postpartum preeclampsia, with subsequent identification of unruptured paraclinal ICA aneurysms. Eleven days after symptom onset, a chiropractic physician was consulted. Based on clinical suspicion, the chiropractor recommended emergent cervical MRA, which revealed a VAD.

The diagnostic delay and lack of cervical vascular imaging before chiropractic evaluation underscore the diagnostic challenges of cervical artery dissection CeAD in postpartum patients. This case reinforces findings in the literature regarding the frequent under-recognition of CeAD in this population [10].

A case series of six pregnant women with postpartum CeAD reported a mean time to diagnosis of 11 days (SD=8), placing the delay in the current case within the previously published range [10]. In the present case, the initial attribution of symptoms to preeclampsia was justifiable, given that neck pain, visual disturbance, and dizziness may occur in this disorder [13]. However, the persistence of symptoms despite preeclampsia treatment represents a critical inflection point, in which further vascular investigation is warranted. The incidental finding of bilateral paraclinoid internal carotid artery aneurysms on brain MRA likely confused the clinical picture, contributing to the delay. Finally, additional delay may have stemmed from a lack of communication of critical MRA findings to the patient.

Despite its potential to cause stroke, CeAD is also occasionally misdiagnosed in non-pregnant people, with a study of 7,090 patients reporting a 3.1% initial misdiagnosis rate in emergency settings, often due to symptoms being benign neurological conditions [7]. The present case underscores the need for heightened suspicion and prompt cervical vascular imaging in postpartum patients with neurologic symptoms of CeAD, such as headache, neck pain, and dizziness.

This patient was initially found to have small, unruptured, bilateral paraclinoid ICA aneurysms. These aneurysms, located in the anterior circulation, may be detected incidentally during neurovascular imaging and are typically asymptomatic unless rupture occurs [14]. While visual disturbances may occur due to the proximity of paraclinoid ICA aneurysms, occipital headache, neck pain, and gait abnormalities are not characteristic of unruptured ICA aneurysms [14]. Instead, these are more commonly associated with posterior circulation events, such as VAD [2].

While the ICA aneurysms were not likely directly responsible for the patient’s acute symptoms, their presence may represent part of a systemic vascular vulnerability that contributed to the development of VAD. In this case, the identification of bilateral aneurysms in a young postpartum patient raises the possibility of an underlying connective tissue disorder, which is a risk factor for both intracranial aneurysm and CeAD [15].

Recently, a retrospective cohort study reported that systemic lupus erythematosus was a risk factor for CeAD [5]. In the present case, mixed connective tissue disease represents a similar condition that can likewise adversely affect blood vessel integrity. In a systematic review of 57 CeAD cases occurring during pregnancy or postpartum, 10% of patients had a known connective tissue disorder [15].

The present case highlights the potential convergence of several risk factors for VAD/CeAD. First, pregnancy itself is associated with arterial dissection, with a potentially heightened risk in the postpartum period [8,9]. Second, arterial dissection risk increases among those with preeclampsia [16]. Third, a connective tissue disease likely predisposed the patient to vascular pathology [5]. Fourth, the patient had a remote history of migraines, which may have been associated with increased CeAD risk [4]. Accordingly, clinicians must be aware of the potentially compounding risk factors for VAD alongside the clinical signs and symptoms. Taken together, a thorough history and exam could prompt earlier vascular imaging and speed the diagnosis of VAD/CeAD.

The earlier chiropractic neck manipulation warrants careful contextual analysis. A history of connective tissue disorder, pregnancy, and recent childbirth warrants consideration, as these factors may represent relative risk factors for spinal manipulative therapy.(SMT). Although some literature suggests an association between cervical manipulation and VAD, high-quality evidence does not support a causal relationship [17]. Importantly, this patient did not experience acute headache, dizziness, or neurological symptoms that may or may not be present in this cohort immediately following the manipulation. These symptoms emerged only postpartum, coinciding with the onset of preeclampsia.

Neuroradiological findings further support a non-causal relationship. Neck MRA demonstrated imaging characteristics consistent with early to late subacute intramural hematoma. Based on signal hyperintensity on a T1-weighted fat-saturated sequence, a consulting neuroradiologist concluded with reasonable medical certainty that the dissection occurred within the two weeks preceding imaging. This timeframe places the vascular event well after the chiropractic intervention performed four weeks earlier [12].

The presence of classic VAD symptoms during this interval further corroborates the radiologic timing, supporting the conclusion that the dissection developed in the immediate postpartum period rather than as a delayed consequence of prior manipulation [2].

Clinicians should adopt a systematic approach to postpartum patients presenting with neurologic symptoms to avoid diagnostic delay and reduce the risk of serious outcomes like stroke [9]. Clinicians should maintain a high index of suspicion for VAD in patients with severe posterior headache, neck pain, and/or neurologic deficits, particularly those with risk factors such as pregnancy, preeclampsia, connective tissue disorders, or other CeAD risk factors (e.g., hypertension, migraine).

A thorough history and neurologic examination that identifies red flag symptoms such as visual disturbances and dizziness should prompt emergent vascular imaging, including the neck (e.g., MRA or computed tomography angiography) [15]. Multidisciplinary collaboration, including input from chiropractors or other outpatient clinicians who recognize neurovascular red flags, is also relevant to timely diagnosis and avoiding stroke.

Strengths and limitations

This case report is supported by detailed imaging findings, a timeline of events, and a multidisciplinary author team representing chiropractic and neuroradiology. However, the single-case design and potential for recall bias limit its generalizability. Future work could expand on this subject by including case series, cohort studies, and case-control designs related to postpartum VAD. These larger studies could improve the understanding of the risk factors and timing for postpartum VAD and facilitate its early detection.

## Conclusions

This case report demonstrates the clinical complexity of VAD in the postpartum period and the potential for diagnostic oversight when initial symptoms are attributed solely to obstetric complications such as preeclampsia. In the present case, a diagnosis of VAD was delayed for two weeks until a chiropractor identified red flag neurovascular factors and advocated for urgent neck MRA, which confirmed the diagnosis. VAD, ischemic events, and impending stroke should remain important differential considerations in postpartum patients who present with headache, neck pain, dizziness, or neurological symptoms, especially in those with connective tissue disorders or other predisposing vascular conditions. Early vascular imaging, including the neck, is essential to avoid missed or delayed diagnoses that may lead to stroke. This case highlights the potential role of chiropractic clinicians in the early recognition of VAD.
